# Does the risk of hypersensitivity reactions to iopromide differ by sex, race, or across regions/countries? An analysis of 152 233 patients from 4 observational studies and the company’s pharmacovigilance database

**DOI:** 10.1093/bjr/tqae190

**Published:** 2024-09-23

**Authors:** Jan Endrikat, Alexander Ullmann, Christoph Gerlinger, Aasia Bhatti, Philipp Lengsfeld, Alexander Michel

**Affiliations:** Radiology R&D, Bayer AG, 13353 Berlin, Germany; Department of Gynecology, Obstetrics and Reproductive Medicine, University Medical School of Saarland, 66421 Homburg/Saar, Germany; Statistics & Data Insights, Bayer AG, Wuppertal, 42113, Germany; Department of Gynecology, Obstetrics and Reproductive Medicine, University Medical School of Saarland, 66421 Homburg/Saar, Germany; Statistics & Data Insights, Bayer AG, Wuppertal, 42113, Germany; Benefit Risk Management Pharmacovigilance, Bayer US LLC, Whippany NJ 07981, United States; Radiology R&D, Bayer AG, 13353 Berlin, Germany; Division Pharmaceuticals, Integrated Evidence Generation, Bayer Consumer Care AG, 4052 Basel, Switzerland

**Keywords:** Safety, hypersensitivity reactions, iopromide, sex, race, region/country

## Abstract

**Objective:**

To analyse the potential impact of patients’ sex, race, and region/country on the risk of hypersensitivity reactions after intra-venous or intra-arterial administration of iopromide.

**Methods:**

Two analyses were performed. (1) The “Phase-IV-Analysis” evaluated an integrated pooled database of 4 non-interventional studies. (2) The “GPV-Analysis” evaluated case reports from the company’s pharmacovigilance database. The Phase-IV-Analysis was a nested case–control analysis of patients who received an injection of iopromide 300/370 mg iodine/mL. Cases had typical/unequivocal HSRs as defined by the ACR Committee on Drugs and Contrast Media 2018. The global pharmacovigilance (GPV)-Analysis was based on HSR case reports in the company database. Exposure estimates were derived from sales/market research data.

**Results:**

The Phase-IV-Analysis comprised 152 233 patients from 37 countries. In the full-analysis set 145 033, 59 412, and 146 649 patients were included in the sex, race, and region/country cohort, respectively. The GPV-Analysis was based on 78.72 million administrations for sex and 118.56 million administrations for region/country. No GPV exposure data by race were available. **Sex**: Phase-IV-Analysis: The HSR incidence was significantly higher for women (0.72%) vs men (0.55%) (*P* ≤ .0001). The unadjusted odds ratio (OR) was 1.3 (95% CI, 1.154-1.499), the adjusted OR was 1.156 (95% CI, 1.006-1.328) (*P* = .04). GPV-Analysis: Reporting rates were 0.0102% for women and 0.0075% for men (*P* < .0001). OR: 1.36 (95% CI, 1.3-1.43). **Race**: Phase-IV-Analysis: No significantly different HSR incidences for white (0.70%) and Asian (0.61%) patients (*P* = .3094) were detected. **Region/country**: Phase-IV-Analysis: The overall world HSR-incidence was 0.62%. Europe: 0.52%, Asia: 0.70%, United States: 0.75%, Germany: 0.51%, China: 0.41%, South Korea: 0.76%. GPV-Analysis: The overall world HSR-reporting rate was 0.015%, varying across regions/countries.

**Conclusion:**

Women showed a slightly higher risk for HSRs than men. Impact of race was not found. HSR-reporting varied by region/country.

**Advances in knowledge:**

Risk for HSRs was increased by female sex but not by race or region/country.

## Introduction

Iopromide (Ultravist) is a low osmolar x-ray contrast medium (LOCM). Iodine is the active ingredient,^1^ which attenuates x-rays in contrast-enhanced computed tomography (CT) and other x-ray-based examinations or interventions.

Iopromide received regulatory approval in February 1985. Over 333 million administrations have been administered to patients in more than 118 countries by December 31, 2022 (data on file).

A number of studies has shown the overall safety profile of iopromide.^2–7^ A few questions on the safety of LOCMs like iopromide^8^ remain to be fully understood. This is particularly true for hypersensitivity reactions (HSR). HSRs are adverse reaction to LOCMs, which can be serious and—in very rare cases—even life-threatening.^9^^,^^10^ However, HSRs are still a hot topic and relevant for the daily routine contrast-enhanced CT imaging.^11^^,^^12^ Iopromide in particular has been investigated in numerous studies,^2–5^ while data on HSRs are scarce.^9^^,^^13^

Using the cohort investigated here, Endrikat et al. previously analysed the HSR risk by administration route^9^ and age.^13^ A potential impact of sex, race, or region/country on HSR risk has not been subject of extensive clinical research yet. It was, however, hypothesized that a different immune status of men and women might impact HRS risk. There is already some evidence that adverse events (AEs) to iodinated contrast media are more common in women compared to men^14–16^ and that there are country-specific risk differences.^14^^,^^17^ For race^18^^,^^19^ and region/country, the literature is scarce.^14^^,^^17^

The objective of this study is to analyse the risk of HSRs to iopromide in patients of different sexes, races, and from different regions/countries.

## Methods

### Databases

This project was based on 2 different data sources: The “Phase-IV-Analysis” leveraged pooled data from 4 Phase-IV observational studies. The “GPV-Analysis” utilized data from the company’s global pharmacovigilance (GPV) database. These databases were already used in 2 previous studies.^9^^,^^13^

#### Phase-IV-Analysis

Four studies on iopromide performed by the company in the past provided a total of 152 233 patients. PMS I (*n* = 74 717)^2^; IMAGE (*n* = 44 835)^4^; Ultravist in CT (*n* = 15 168)^20^; and TRUST (*n* = 17 513).^21^ These studies were conducted in accordance with the Declaration of Helsinki. Ethics Committees’/Institutional Review Boards’ approvals, and patient informed consent forms were collected and stored in the study centres of the countries. As this project is considered a voluntary Post-Authorization Safety Study (PASS), it has been registered at ClinicalTrials.gov (NCT05428397) and at ENCePP (EUPAS47805).

#### GPV-Analysis

The company’s pharmacovigilance records AEs from spontaneous reporting and other sources. Exposure estimates were derived from sales data and data from market research.^22^ However, while total exposure was ˃333 million by December 31, 2022, exposure estimates by sex or region/country were only available for selected countries and years. No exposure estimates by race were available.

The HSR risk by sex was calculated based on 78.72 million administrations in 10 countries between January 2013 and December 2020, based on sales and market research data.

HSR risk by region/country was calculated on 118.56 million administrations in 37 countries between January 2011 and December 2022, based on sales data.

### Study population

#### Phase-IV-Analysis

Patients of all age groups received either an intra-venous or intra-arterial injection of iopromide with either 300 or 370 mg iodine/mL for various indications.

#### GPV-Analysis

The GPV data are covering patients of all ages, receiving iopromide in all indications with a wide range of dosing and different routes of administration.

### Definition of cases and controls

#### Phase-IV-Analysis

Cases were defined as patients with a typical and unequivocal HSR as defined by the ACR Committee on Drugs and Contrast Media 2018; Version 10.3.^23^ Irrespective of the investigators’ assessment, all cases were categorized as drug related, that is, always the most conservative approach for drug relationship was chosen.

Controls were defined as patients in which no AE was reported. Unspecific reactions (eg, headache, nausea) and possibly procedure-related reactions (eg, drop in blood pressure, bradycardia, tachycardia) were excluded from the cases and from the controls to avoid misclassification and confounding by the procedure performed.

AE data were coded by Medical Dictionary for Regulatory Activities (MedDRA) version 25.0.

#### GPV-Analysis

The pharmacovigilance database includes cases retrieved according to retrieval criteria (MedDRA Query Immediate Type Hypersensitivity criteria).

### Statistics

#### Phase-IV-Analysis

Statistical analyses were exploratory, without confirmatory hypothesis testing. Any *P*-values extracted were seen as indicators of uncertainty, without multiplicity adjustment.

Statistical methods were used descriptively for all variables: categorical variables using frequency tables, and continuous variables using sample statistics, such as mean, SD, and quantiles. Continuous variables were portrayed both as absolute value and change from baseline when applicable.

The statistical results were based on 4 main variables: sex, race, geographical region, and country. Each of these variables was subjected to statistical calculations based on subjects with non-missing data sets. The full analysis set included patients who received an iopromide injection of 300 or 370 mg/mL and had recorded values for sex, race, region, or country. In contrast, the complete cases analysis set comprised of patients from the full set who also had no missing data within the pre-specified list of potential confounders.

We calculated adjusted and unadjusted odds ratios (ORs) with 95% CI for risk factors: sex, race, country, and geographical region via multivariate logistic regression models. The models were adjusted for the aforementioned factors, integrating potential confounders, and consistently included age, study effect, and sex.

Variable selection was guided by the effect’s importance, denoted by a descriptive *P*-value. A confounder with a Wald test *P*-value below 0.1 in a logistic regression model was deemed significant. Covariates identified important by these adjusted models were combined in a multivariate logistic regression model to pinpoint individual effects on HSRs. This analysis was performed separately for each primary variable of interest. Unadjusted incidences were calculated on full analysis set (FAS) using Fisher’s Exact Test to obtain *P*-values.

#### GPV-Analysis

The reporting rate for HSRs was calculated only for those countries and years for which sales data per sex and/or per country where available. The reporting rate for HSRs was calculated by dividing the sum of all HSRs in a group by the number of total administrations in this group times 100%. The null hypothesis of equal reporting rates between groups was exploratively tested using the Cochran–Mantel–Haenszel chi-squared test controlling for country with continuity correction at a comparison-wise significance level of 5%.

## Results

### Disposition of patients

#### Phase-IV-Analysis

**Figure 1.**
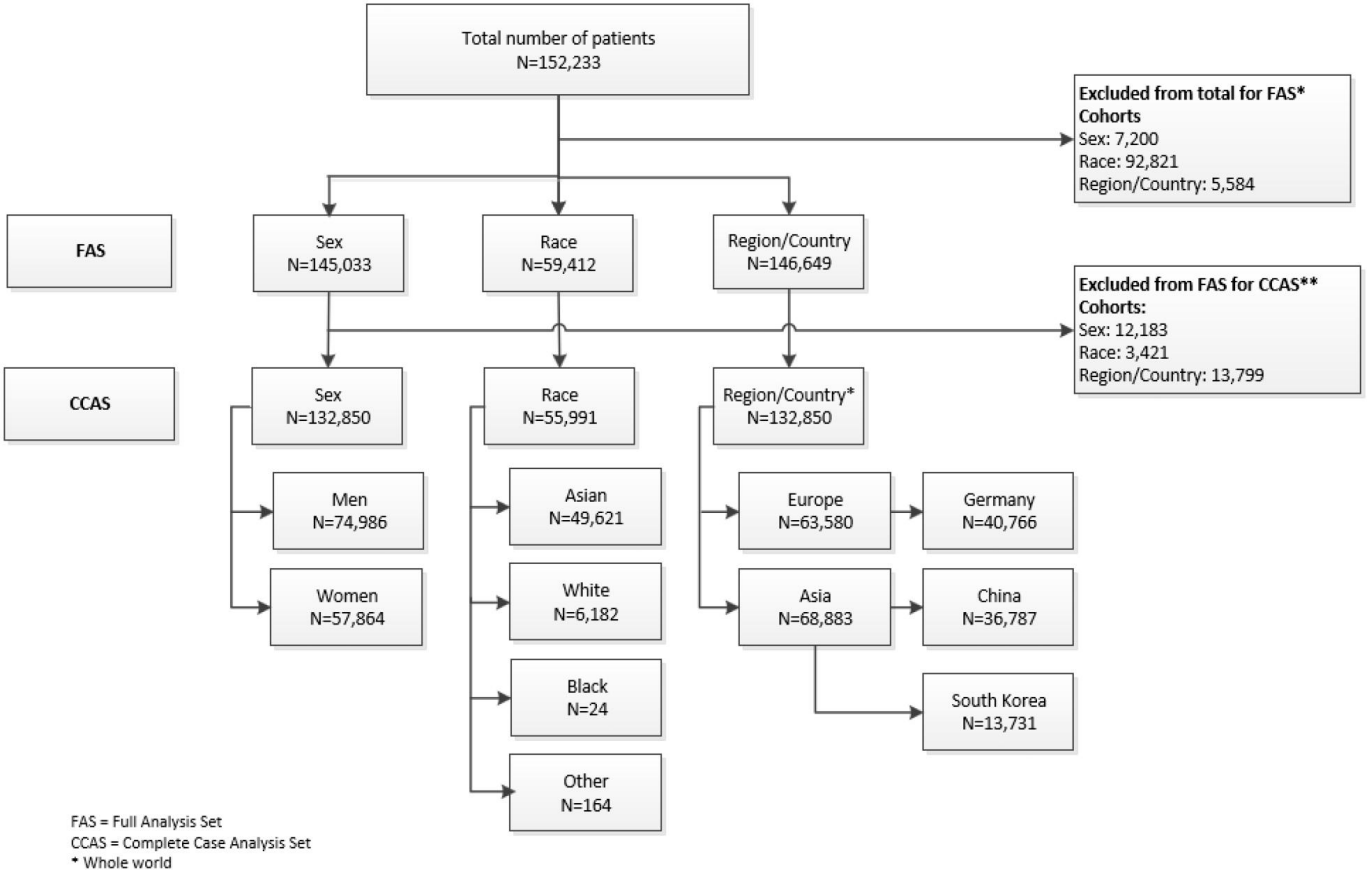
Phase-IV-Analysis—Disposition of Patients. *Reasons for exclusion form FAS: (1) No injection of iopromide 300/370 mg/mL. (2) Not deemed as either a case or a control for the primary analysis. (3) Either no sex, race, or region/country recorded (multiple exclusion criteria were possible). **Reasons for exclusion form CCAS: (4) No age recorded. (5) Injection route not recorded. (6) Contrast medium dose (volume) not recorded. (7) Dose of iodine in contrast medium not recorded. (multiple exclusion criteria were possible). CCAS = complete case analysis; FAS = full analysis set.

Overall, 152 233 patients had been recruited across the 4 observational studies. Patients lacking data on either sex, race, or region/country as well as confirmation of iopromide injection, or patients otherwise not eligible for primary analysis were excluded from FAS. Patients lacking information on age, injection route, iopromide volume, or iodine dose were excluded from complete case analysis (CCAS). The CCAS comprised 132 850 patients for sex, 55 991 patients for race and 132 850 for region/country. The patient numbers for the 3 evaluations are shown in Figure 1.

#### GPV-Analysis

For the evaluation on sex, reports covering 78.72 million administrations between 2013 and 2020 from Brazil, France, Germany, Italy, South Korea, China Russia, Spain, the United States, and Great Britain were included. Exposure data were not available by race, and, therefore, race from GPV data were not analysed.

**Figure 2.**
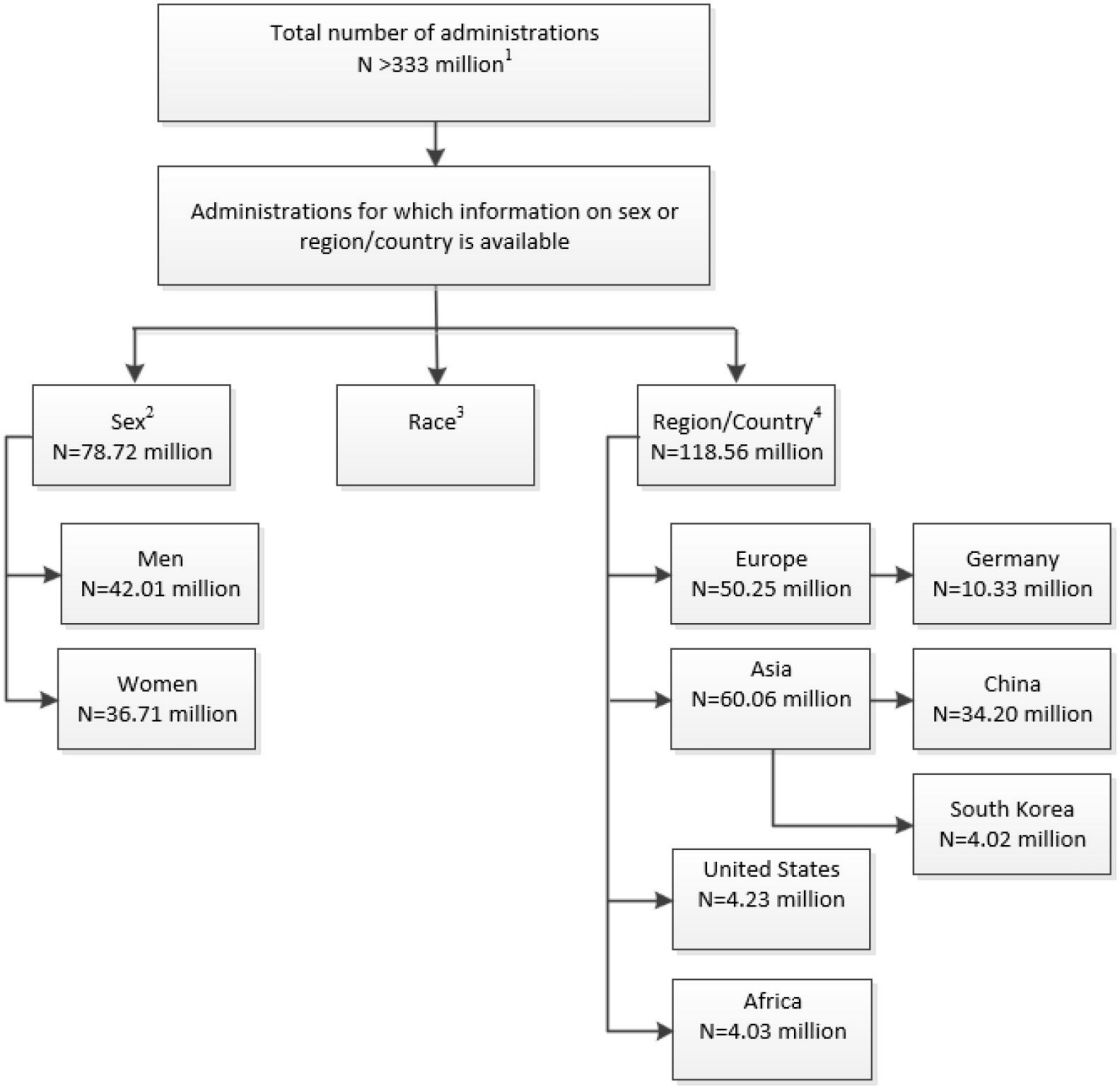
GPV-Analysis—administrations. ^1^Data from January 1995 till December 2022; 118 countries. ^2^Data from January 2013 till December 2020; 10 countries. ^3^For race no sales or market research data was available. ^4^Data from January 2011 till December 2022; 37 countries. GPV = global pharmacovigilance.

For the evaluation of country and regions ˃118.56 million administrations from 37 countries were analysed (Figure 2).

### Demographics of study population

#### Phase-IV-Analysis

The demographics of the sex and region cohorts were almost identical (Table 1). The demographics of the considerably smaller race cohort differed from the other 2 cohorts in many aspects: less patients from Europe and more from China, less iopromide 300 and more iopromide 370 injections, more Asian and white patients, more frequent arterial hypertension and corticosteroid administration, less intra-venous and more intra-arterial administration, more abdominal, cardiac, and thorax examinations, and—finally—more imaging for tumours and pain (Table 1).

**Table 1.** Demographics of study population of Phase-IV-Analysis (FAS).

Total (*N* = 152 233)	Sex (*N* = 145 033); 100%	Race (*N* = 59 412); 100%	Region (*N* = 146 649); 100%
Age (years), mean (SD)	55.9 (16.0)	55.6 (14.9)	55.9 (16.0)
Geographic region			
Europe	68 184 (47.0%)	9021 (15.2%)	69 561 (47.4%)
Asia (excl. China)	32 779 (22.6%)	13 477 (22.7%)	32 971 (22.5%)
China	36 905 (25.4%)	36 914 (62.1%)	36 928 (25.2%)
North America	6770 (4.7%)	—	6791 (4.6%)
Africa	395 (0.3%)		398 (0.3%)
Concentration^a^			
Iopromide 300	93 274 (64.3%)	24 536 (41.3%)	94 529 (64.5%)
Iopromide 370	51 759 (35.7%)	34 876 (58.7%)	52 120 (35.5%)
Sex			
Men	80 816 (55.7%)	35 528 (59.8%)	80 816 (55.1%)
Women	64 217 (44.3%)	23 768 (40.0%)	64 217 (43.8%)
Not specified	—	116 (0.2%)	1616 (1.1%)
Race			
Asian	50 164 (34.6%)	50 217 (84.5%)	50 217 (34.2%)
White	8921 (6.2%)	8984 (15.1%)	8984 (6.1%)
Black	30 (<0.1%)	30 (0.05%)	30 (<0.1%)
Other	181 (0.1%)	181 (0.3%)	181 (0.1%)
Not specified	85 737 (59.1%)		87 237 (59.5%)
Concomitant disease			
Hypertension arterial	17 503 (12.1%)	15 988 (26.9%)	17 579 (12.0%)
Coronary heart disease	12 367 (8.5%)	4719 (7.9%)	12 515 (8.5%)
Diabetes mellitus	11 275 (7.8%)	5913 (10.0%)	11 388 (7.8%)
Reduced general condition	7340 (5.1%)	4475 (7.5%)	7413 (5.1%)
Specific contrast media risk factor	5746 (4.0%)	2045 (3.4%)	5818 (4.0%)
Allergy	4262 (2.9%)	1301 (2.2%)	4315 (2.9%)
Asthma	907 (0.6%)	587 (1.0%)	917 (0.6%)
Contrast media reaction	795 (0.5%)	227 (0.4%)	804 (0.5%)
Other	21 645 (14.9%)	12 188 (20.5%)	21 866 (14.9%)
None specified	26 657 (18.4%)	26 515 (44.6%)	26 799 (18.3%)
Pre-medication			
Corticosteroids	10 681 (7.4%)	8537 (14.4%)	10 743 (7.3%)
H1/H2 blocker	3640 (2.5%)	472 (0.8%)	3698 (2.5%)
H1/H2 blocker or corticosteroids	35 (<0.1%)		36 (<0.1%)
Other	6007 (4.1%)	2938 (4.9%)	6076 (4.1%)
None specified	268 (0.2%)	237 (0.4%)	273 (0.2%)
Injection route			
Intravenous	115 914 (79.9%)	38 806 (65.3%)	117 343 (80.0%)
Intra-arterial	28 331 (19.5%)	20 419 (34.4%)	28 489 (19.4%)
Other	397 (0.3%)	99 (0.2%)	410 (0.3%)
Not specified	302 (0.2%)	—	316 (0.2%)
Missing	89 (<0.1%)	88 (0.1%)	91 (0.1%)
Examination region^b^			
Abdomen	26 873 (18.5%)	18 989 (32.0%)	27 074 (18.5%)
Cardiac/cardiac vessels	23 017 (15.9%)	22 867 (38.5%)	23 058 (15.7%)
Thorax	13 992 (9.6%)	9448 (15.9%)	14 101 (9.6%)
Pelvis	8197 (5.7%)	4917 (8.3%)	8263 (5.6%)
Head/brain	6664 (4.6%)	4273 (7.2%)	6726 (4.6%)
Kidney/renal vessels	4702 (3.2%)	3783 (6.4%)	4742 (3.2%)
Neck	2702 (1.9%)	1835 (3.1%)	2717 (1.9%)
Blood vessels	1951 (1.3%)	1668 (2.8%)	1967 (1.3%)
Limbs	414 (0.3%)	356 (0.6%)	415 (0.3%)
Joints	46 (<0.1%)	25 (<0.1%)	46 (<0.1%)
Other	1133 (0.8%)	1019 (1.7%)	1148 (0.8%)
Not specified	9 (<0.1%)	9 (<0.1%)	20 (<0.1%)
Missing	71 107 (49.0%)		72 289 (49.3%)
Indication			
Tumor/suspicion of tumor	26 460 (18.2%)	20 672 (34.8%)	26 620 (18.2%)
Pain	7543 (5.2%)	5466 (9.2%)	7597 (5.2%)
Post-therapy-control	7526 (5.2%)	5260 (8.9%)	7588 (5.2%)
Staging	5458 (3.8%)	3629 (6.1%)	5510 (3.8%)
Inflammatory diseases	4378 (3.0%)	3001 (5.1%)	4407 (3.0%)
Infarct/suspicion of infarct	3464 (2.4%)	3062 (5.2%)	3488 (2.4%)
Haemorrhage	948 (0.7%)	763 (1.3%)	954 (0.7%)
Trauma	616 (0.4%)	382 (0.6%)	618 (0.4%)
Other	24 496 (16.9%)	22 631 (38.1%)	24 596 (16.8%)
Not specified	59 (<0.1%)	62 (0.1%)	77 (<0.1%)
Missing	71 546 (49.3%)		72 736 (49.6%)
Iodine dose			
≤20 g	24 213 (16.7%)	9851 (16.6%)	24 467 (16.7%)
>20-40 g	94 371 (65.1%)	38 120 (64.2%)	95 496 (65.1%)
>40-60 g	19 393 (13.4%)	6660 (11.2%)	19 549 (13.3%)
>60 g	6413 (4.4%)	4711 (7.9%)	6453 (4.4%)
Not specified	643 (0.4%)	70 (0.1%)	684 (0.5%)
Type of examination			
CT	64 660 (44.6%)	8(<0.1%)	65 569 (44.7%)
CT (multi slice)	33 118 (22.8%)	33 005 (55.6%)	33 240 (22.7%)
Angiocardiography	12 555 (8.7%)	12 170 (20.5%)	12 582 (8.6%)
Urography	10 683 (7.4%)		10 975 (7.5%)
CT (single slice)	3979 (2.7%)	3896 (6.6%)	4037 (2.8%)
Angiography	1855 (1.3%)	789 (1.3%)	1881 (1.3%)
Phlebography	326 (0.2%)	325 (0.5%)	330 (0.2%)
DSA	263 (0.2%)	207 (0.3%)	264 (0.2%)
PTCA	176 (0.1%)		179 (0.1%)
PTA	83 (<0.1%)		84 (<0.1%)
Other	6992 (4.8%)	7013 (11.8%)	7013 (4.8%)
Not specified	10 343 (7.1%)	1999 (3.4%)	10 495 (7.2%)

a It was not checked if iopromide has been diluted.^39^

b Multiple reasons possible.

Abbreviation: CT = computed tomography; DSA = digital subtraction angiography; FAS = full analysis set; PTCA = percutaneous transluminal cardiac angioplasty; PTA = percutaneous transluminal angioplasty.

#### GPV-Analysis

Patients’ age groups were predominantly adults (62.7% of the cases) and elderly (21.9%) (cumulative data till December 31, 2022).

### Analysis of HSR incidence and reporting rates by sex

#### Phase-IV-Analysis

**Figure 3.**
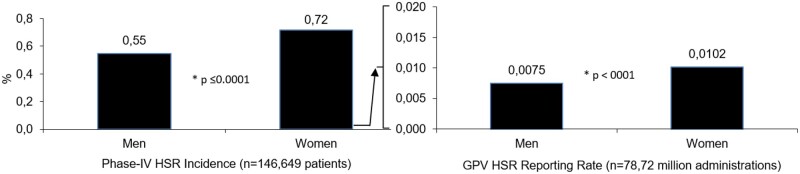
Incidence and reporting rates of HSRs by sex (FAS). FAS = full analysis set; HSR = hypersensitivity reaction.

**Figure 4.**
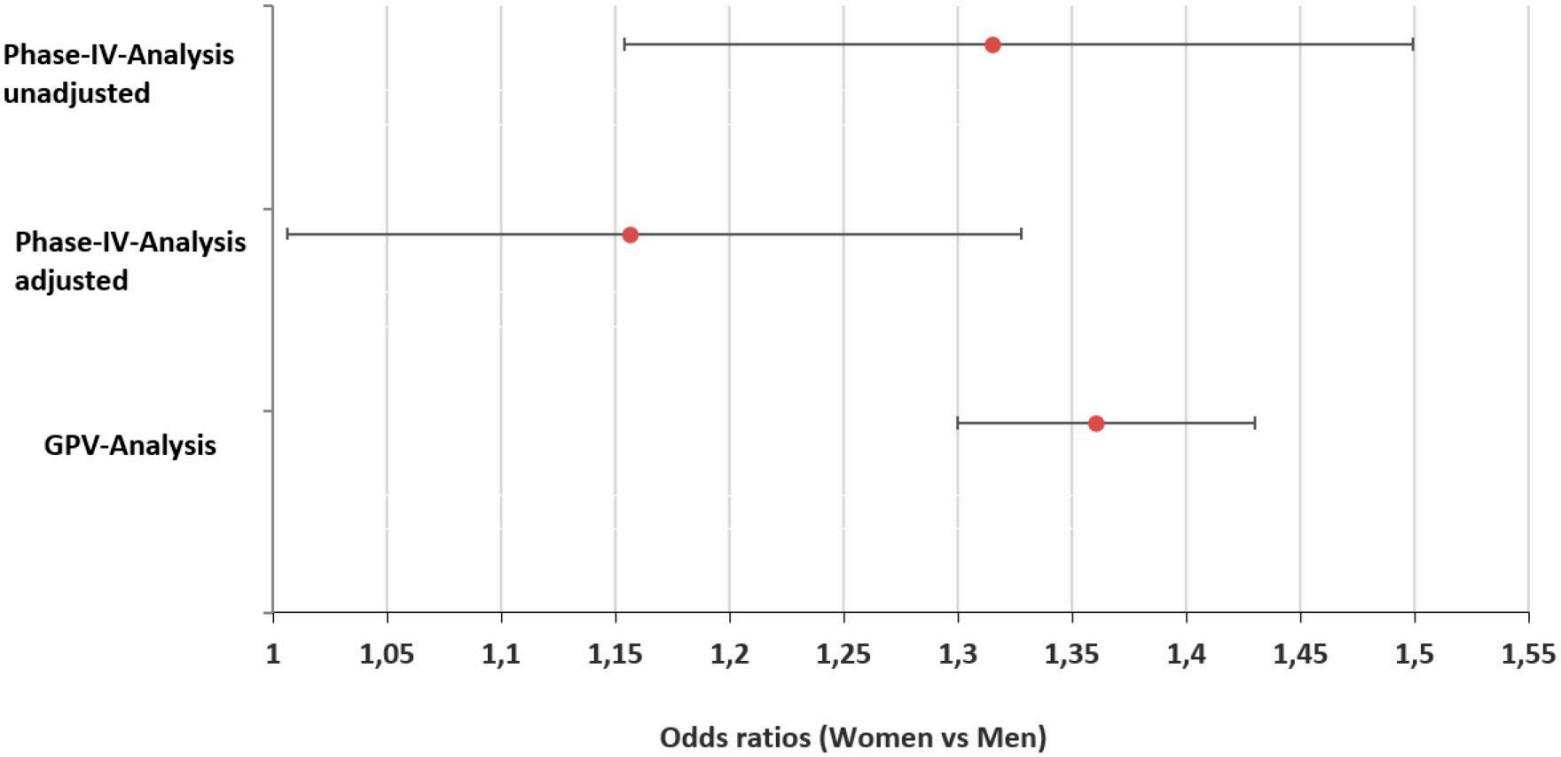
Odds ratios for HSRs in women and men in Observational Studies and Global Pharmacovigilance Database. Phase-IV-Analysis: odds ratio and 95% CI before and after adjusting for possible confounding covariates (Table 4). GPV-Analysis: odds ratios and 95% CI are derived from a Mantel–Haenszel test controlling for country. GPV = global pharmacovigilance; HSR = hypersensitivity reaction.

In the FAS, the HSR incidence was significantly higher for women (461/64 217 = 0.72%) compared to men (442/80 816 = 0.55%) (*P* ≤ .001) (Table 2, Figure 3). This result was confirmed by the unadjusted and adjusted ORs. The unadjusted OR based on FAS was 1.315 (95% CI, 1.154-1.499), the adjusted OR based on CCAS was 1.156 (95% CI, 1.006-1.328) for women (*P* = .0409) (Figure 4, Table 3).

**Table 2.** Incidence of HSRs in Phase-IV-Analysis (FAS).

		Cases	Controls	Total	Incidence^a^ (%)	*P*-value, Fisher’s exact test
Sex	Men	442	80 374	80 816	0.55	
	Women	461	63 756	64 217	0.72	≤.0001
Race	White	63	8921	8984	0.70	
	Asian	307	49 910	50 217	0.61	.3094
	White	63	8921	8984	0.70	
	Other	8	173	181	4.42	≤.0001
Region	Europe	364	69 197	69 561	0.52	
	ROW	541	76 547	77 088	0.70	≤.0001
	Asia	490	69 409	69 899	0.70	
	ROW	415	76 335	76 750	0.54	≤.0001
	United States	51	6740	6791	0.75	
	ROW	854	139 004	139 858	0.61	.1527
	Germany^b^	234	45 384	45 618	0.51	
	ROW	671	100 360	101 031	0.66	.0005
	China^b^	152	36 776	36 928	0.41	
	ROW	753	108 968	109 721	0.69	≤.0001
	South Korea^b^	105	13 640	13 745	0.76	
	ROW	800	132 104	132 904	0.60	.0254

Abbreviation: FAS = full analysis set; ROW = rest of the world.

a In the Phase-IV-Analysis, % of patients with HSR is called “incidence,” as the diagnosis is confirmed by a healthcare professional. In the GPV-Analysis, % of HSR cases per administrations is called “reporting rate,” as patients might have gotten multiple doses of iopromide.

b Countries with more than 10 000 patients.

**Table 3.** Cases/controls/adjusted odds ratios (CCAS).

		Cases	Controls	Total	Odds ratios (95% CI)	Wald *P*-value
Sex	Men	411	74 575	74 986	1.000	.0409
	Women	407	57 457	57 864	1.156 (1.006-1.328)	
Race	White	48	6134	6182	1.000	<.0001
	Asian	301	49 320	49 621	0.972 (0.704-1.344)	
Region	Europe	341	63 239	63 580	0.644 (0.543-0.765)	<.0001
	ROW	477	68 793	69 270	1.000	
	Asia	477	68 406	68 883	1.596 (1.344-1.897)	<.00001
	ROW	341	63 626	63 967	1.000	
	United States	51	6716	6767	1.117 (0.829-1.504)	.4683
	ROW	846	136 001	136 847	1.000	
Country	Germany	213	40 553	40 766	0.692 (0.574-0.835)	.0001
	ROW	605	91 479	92 084	1.000	
	China	151	36 636	36 787	0.692 (0.554-0.864)	.0012
	ROW	667	95 396	96 063	1.000	
	South Korea	105	13 626	13 731	1.239 (1.003-1.531)	.0467
	ROW	713	118 406	119 119	1.000	

Abbreviation: CCAS = complete case analysis; ROW = rest of the world.

The important covariates that were adjusted for to show the independent effect of sex are shown on Table 4.

**Table 4.** Sensitivity analysis.

Covariate	Covariate Level	Sex	Sex	Race	Race	Region	Region
		Odds Ratio (95% CI)	Wald *P*-value	Odds ratio (95% CI)	Wald *P*-value	Odds ratio (95% CI)	Wald *P*-value
Sex	Male	1.000	.0409	1.000	.0595	1.000	.0289
	Female	1.156 (1.006-1.328)		1.225 (0.992-1.512)		1.168 (1.016-1.342)	
Race^a^	Asian	—	—	0.972 (0.704-1.344)	<.0001		
	White	—	—	1.000			
Region	APAC	—	—	—	—	1.665 (1.383-2.006)	<.0001
	Europe	—	—	—	—	1.000	
	Rest of world		—	—	—	1.334 (1.080-1.648)	
Age group (years)	<18	1.114 (0.643-1.930)	<.0001	1.391 (0.683-2.835)	.0004	1.054 (0.607-1.827)	<.0001
	≥18 and < 50	2.212 (1.825-2.680)		1.942 (1.424-2.649)		2.126 (1.752-2.580)	
	≥50 and < 65	1.700 (1.403-2.060)		1.737 (1.276-2.365)		1.668 (1.377-2.022)	
	≥65	1.000		1.000		1.000	
Study	IMAGE	1.000	<.0001	1.000	<.0001	1.000	<.0001
	TRUST	0.145 (0.078-0.268)		0.161 (0.067-0.388)		0.141 (0.074-0.266)	
	PMS I	0.669 (0.570-0.786)		—	—	0.839 (0.690-1.020)	
	Ultravist in CT	0.839 (0.672-1.047)		—	—	1.045 (0.826-1.323)	
Injection route	Intra-venous	1.000	.0081	1.000	.2473	1.000	.0082
	Intra-arterial	0.615 (0.429-0.881)		0.651 (0.314-1.348)		0.615 (0.429-0.882)	
CAD	No	1.000	.0780	1.000	.0126	1.000	.0909
	Yes	0.736 (0.524-1.035)		0.488 (0.277-0.857)		0.745 (0.530-1.048)	
Hypertension	No	—	—	—	—	1.000	.2139
	Yes	—	—	—	—	1.195 (0.902-1.582)	
Reduced condition	No	—	—	1.000	.0002		
	Yes	—	—	0.379 (0.229-0.628)			
Diabetes mellitus	No	1.000	.0009	—	—	1.000	.0021
	Yes	1.596 (1.211-2.104)		—	—	1.545 (1.171-2.038)	
Allergy	No	1.000	<.0001	1.000	<.0001	1.000	<.0001
	Yes	3.491 (2.669-4.566)		3.358 (2.141-5.265)		3.565 (2.718-4.676)	
Asthma	No	1.000	.0075	—	—	1.000	.0104
	Yes	2.070 (1.214-3.529)		—	—	2.013 (1.178-3.439)	
CM reaction	No	1.000	<.0001	1.000	<.0001	1.000	<.0001
	Yes	4.161 (2.642-6.556)		6.966 (2.945-16.475)		4.289 (2.716-6.771)	
Other concomitant disease	No	1.000	.0192	—	—	1.000	.0174
	Yes	1.300 (1.044-1.620)		—-	—	1.313 (1.049-1.643)	
CM risk factor	No	1.000	.3415	—	—	1.000	.3744
	Yes	1.110 (0.895-1.377)		—	—	1.107 (0.884-1.387)	
Dose (mL)	<100	—	—	1.000	.0137	—	—
	≥100 and <150	—	—	0.688 (0.547-0.866)		—	—
	≥150 and <200	—	—	0.572 (0.248-1.318)		—	—
	≥200 and <300	—	—	0.534 (0.161-1.764)		—	—
	≥300	—	—	0.327 (0.045-2.375)		—	—

Summary of important covariates for HSRs (CCAS).

CAD = coronary artery disease; CCAS = Complete case analysis; CM = contrast medium; CT = computed tomography.

a Black race (*n* = 24) and “other races” (*n* = 164) are not listed because of the low sample size.

#### GPV-Analysis

A total of 6911 cases were reported from a coverage of 78.72 million administrations, 3166 in men and 3745 cases in women (estimated administrations: ˃42.01 million in men, ˃36.71 million in women). This resulted in reporting rates of 0.0075% and 0.0102% in men and women, respectively (*P* < .0001) (Figure 3). The OR for HSR risk for women vs men was 1.36 (95% CI 1.3-1.43) (Figure 4).

### HSR incidence by race

#### Phase-IV-Analysis

**Figure 5.**
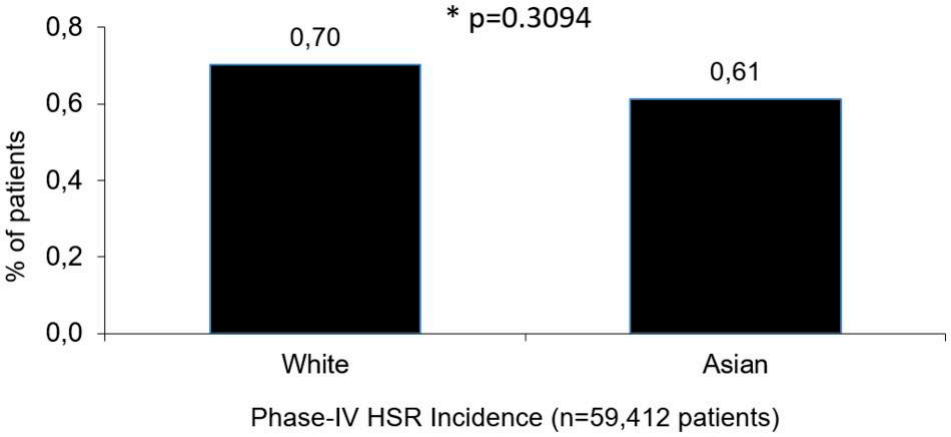
Incidence of HSRs by Race (FAS). FAS = full analysis set; HSR = hypersensitivity reaction.

In the FAS, the HSR incidence did not differ significantly, 0.70% for white patients vs 0.61% for Asians (*P* = .3094) (Table 2, Figure 5).

This finding was confirmed by the adjusted ORs for Asians of 0.972 (95% CI, 0.704-1.344) vs white patients 1.00 (Table 3).

The important covariates that were adjusted for to show the independent effect of race are shown on Table 4.

### HSR incidence and reporting rates by region/country

#### Phase-IV-Analysis

In regions Europe and Asia, the sample size was equally about 69 000 patients. Only Germany, China, and South Korea included >10 000 patients (Table 2, Table 5). The overall world HSR incidence was 0.62% (Table 5).

**Table 5.** Cases/controls/incidence/reporting rate for region/countries (FAS).

		Phase-IV-Analysis			GPV-Analysis 2011-2022^a^		
Region	Country	Total	Cases	Incidence (%)	Cases	Administrations	Reporting rate (%)
Asia	China	36 928	152	0.41	8028	34 203 841	0.0235
	Indonesia	692	15	2.17	123	942 010	0.0131
	Iran	5088	66	1.30	20	2 358 550	0.0008
	Jordan	1668	9	0.54	3	826 670	0.0004
	South Korea	13 745	105	0.76	121	4 016 321	0.0030
	Malaysia	863	7	0.81	157	1 678 259	0.0094
	Pakistan	1398	4	0.29	50	3 273 701	0.0015
	Philippines	4175	41	0.98	90	1 420 994	0.0063
	Saudi Arabia	742	9	1.21	0	273 484	0.0000
	Singapore	1657	52	3.14	71	55 470	0.1280
	Taiwan	768	13	1.69	375	2 393 884	0.0157
	Thailand	562	16	2.85	597	6 687 432	0.0089
	Vietnam	1613	1	0.06	141	1 925 620	0.0073
		69 899	490	0.70	9776	60 056 236	0.0163
Europe	B/Herzegovina	522	0	0.00	77	403 788	0.0191
	Bulgaria	291	1	0.34	10	346 190	0.0029
	Croatia	432	1	0.23	23	182 754	0.0126
	Estonia	173	0	0.00	8	47 672	0.0168
	Finland	443	1	0.23	0	135	0.0000
	Germany	45 618	234	0.51	730	10 334 400	0.0071
	Hungary	219	0	0.00	107	504 197	0.0212
	Italy	695	10	1.44	2502	4 052 395	0.0617
	Latvia	399	0	0.00	12	171 635	0.0070
	Lithuania	2380	1	0.04	5	381 816	0.0013
	Moldovia	358	2	0.56	8	82 487	0.0097
	Poland	2749	31	1.13	1021	5 485 780	0.0186
	Serbia	682	4	0.59	107	906 448	0.0118
	Romania	2397	3	0.13	134	780 689	0.0172
	Russia	614	10	1.63	984	13 012 242	0.0176
	Slovakia	1255	1	0.08	30	569 701	0.0053
	Slovenia	4787	37	0.77	45	187 898	0.0239
	Spain	1906	8	0.42	1127	3 672 392	0.0307
	Switzerland	1404	12	0.85	221	1 332 691	0.0166
	Turkey	332	2	0.60	53	6 363 788	0.0008
	Ukraine	1905	6	0.31	42	1 429 441	0.0029
		69 561	364	0.52	7246	50 248 539	0.0144
Africa	Egypt	298	0		41	3 231 750	0.0013
	South Africa	100	0		77	799 205	0.0096
		398	0	0.00	118	4 030 955	0.0029
America	United States	6791	51	0.75	701	4 230 935	0.0166
Total	37 countries	146 649	905	0.62	17 841	118 566 665	0.0150

Abbreviations: FAS = full analysis set; GPV: global pharmacovigilance.

a January 1, 2011 to December 31, 2022.

**Figure 6.**
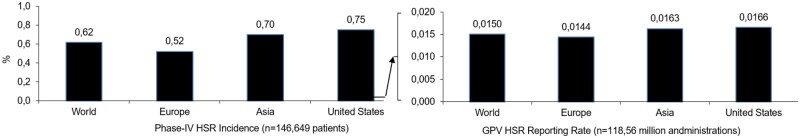
HSR incidence and reporting rates by region (FAS). FAS = full analysis set; HSR = hypersensitivity reaction.

The incidence of 1 region/country was compared with the respective rest of the world (ROW). While the HSR incidence in Germany (0.51%) matched the European result (0.52%) and the HSR incidence in South Korea (0.76%) matched the overall Asia result (0.70%), the value for China (0.41%) was lower than overall Asia (0.70%). Overall, Europe, Germany, and China showed lower HSR incidences, whereas Asia, United States, and South Korea showed higher HSR incidences compared with the respective ROW (Tables 2 and 5, Figure 6).

These results were confirmed by the ORs (Table 3). The important covariates that were adjusted for to show the pure effect of the region are shown on Table 4.

#### GPV-Analysis

The overall world HSR reporting rate was 0.0150. There were lower reporting rates for Europe and higher rates for Asia and the United States compared to the overall world (Table 5, Figure 6). The HSR reporting rates for all countries are shown on Table 5. The HSR reporting rates vary from 0.00% (Saudi Arabia, Finland) to 0.13% (Singapore). All other countries showed reporting rates ≤0.062% (Italy).

## Discussion

This project analysed the risk of HSRs to iopromide in patients of different sexes, races, and from different regions/countries.

### Study design

Based on 2 large databases, 2 different analyses were performed. This approach of using 2 separate data sources with considerably high numbers of patients suffering from HSRs is assumed to provide very solid, statistically supported, results. Although only data on iopromide have been included, these findings should be representative for other LOCMs.

In the Phase-IV-Analysis, the HSR risk is called “incidence,” while in the GPV-Analysis, the risk is called “reporting rate.” Both parameters describe the percentage of HSR cases in the respective cohort. However, data capture in the 4 pooled prospective observational studies, basis of the Phase-IV-Analysis, followed a structured, well-defined protocol. Investigators were trained on recognizing the symptoms and informed about the ACR definitions of HSRs.^11^

In contrast, the GPV-Analysis included spontaneous reports as well as cases from other sources reported by health-care professionals, consumers, and others. They usually focus on reporting more severe cases and tend to underreport the milder ones.^24^ The reporting rates found in the GPV-Analysis were 2 orders of magnitude lower than the incidences found in the Phase-IV-Analysis, well in line with findings reported by other authors.^18^^,^^25–27^

### Sex

In both analyses, the HSR risk was significantly higher for women compared to men (Figure 3). Voltolini et al. reported on 407 patients who developed HSRs after application of various LOCMs and a control group of 152 patients. In the HSR group, 60% were women, in the control group 44%, resulting in an OR for men of 0.5 (95% CI, 0.4-08). They claimed male gender as protective factor or ICM reactions.^16^

Lin et al. evaluated >11.3 million AE reports on 6 iodinated contrast media including iopromide from the US Food and Drug Administration Adverse Event Reporting system. Their study design was close to our GPV-Analysis, although with a smaller cohort. They identified 5432 cases of HSRs: 39% in women, 28% in men, and 33% in patients with unknown sex. While primarily focusing on the HSR risk profile of the 6 different brands, they also analysed 3 specific HSRs by sex and brand. Angioedemas were more frequent in women, severe cutaneous AEs were somewhat balanced, and anaphylactic shocks were more often diagnosed in men. Interestingly, this pattern was seen not only for iopromide but for almost all brands, providing evidence for a class effect.^14^

In summary, the results of the 2 analyses are in line with other studies, showing an association of female gender with increased risk for HSRs.^15^^,^^28^

There are hypotheses about the pathomechanism for this phenomenon. For example, De Martinis et al. suggested that genetic factors linked to chromosome X, epigenetic changes, influences of sex hormones on immune cells, and exposure to medications could be responsible.^29^ This would be in line with the findings of previous studies indicating importance of lung passage^9^ and immune status of patients.^13^

### Race

Compared to white individuals, Asians showed a similar risk for HSRs (Figure 5). An incidence calculation for Black and non-classified (“other”) patients was not reasonable because of the low sample size: No case was reported in 24 Black patients but 8 cases in 164 “other” patients (Figure 1). In addition, an evaluation of race in the GPV-Analysis was impossible.

The question whether or not race—or ethnicity—plays a role in HSR risk has been touched—to the best of our knowledge—only by 2 research groups^18^^,^^19^ so far.

Analyzing HSR risk (“anaphylaxis”), Pagani et al. (see also above) reported an adjusted OR for Caucasians vs “other” of 1.16 (0.93-1.45), which is also not significant. Caucasians made up 80.4% of the total population and 82.3% of the HSR cases. Unfortunately, they did not specify the group of “other.” However, as 10/37 (27%) studies of their review were from Asia, it might be assumed that the “other group” included a considerable number of Asian subjects.^19^ If this assumption holds true, Pagani et al. also did not see any trend for a race dependent HSR susceptibility.

Kim et al. analysed 1969 ADRs in 142 099 Korean patients showing different HSR (“anaphylaxis”) incidences after administration of different LOCMs ranging from 0.012% to 0.041%. While they could not find an effect of sex on HSR incidence (*P* = .142), their findings confirmed other Asian studies, which also evaluated Korean and Japanese patients. As European and North American studies did not find such brand differences, Kim et al. speculated that ethnicity might play a role.^18^

While data on race and HSR risk is scarce, this study adds another piece of evidence on this question, indicating that race seems to have no impact on HSR risk.

### Region/country

The overall world HSR incidence was 0.62% (Phase-IV-Analysis) and the overall world reporting rate was 0.015% (GPV-Analysis) (Figure 6). The reason for the huge difference between the 2 analyses can easily be explained by the different data capture/study designs, as has been explained above.

Both analyses showed small differences between regions with somewhat lower HSRs in Europe and higher in Asia and the United States compared to overall world (Figure 6). In addition, there were also some variations between individual countries.

As the reporting of all AEs was standardized in the 4 Phase-IV-study protocols, the different HSR incidences can be explained by the sample size in each country (Table 5).

The GPV-Analysis is based on mostly spontaneous ADR reporting in each country. It is well established that reporting standards vary between national pharmacovigilance reporting systems.^30–35^

In addition, the national income seems to play a role, as high-income countries seem to have generally higher ADR reporting rates compared to low-income countries.^36^ Both reporting standards and economic status of countries are suggested to sufficiently explain the country differences in HSR reporting rates in the GPV-Analysis.

HSRs to radiocontrast media were also reported by Lee et al. from the Asia Pacific region. They systematically reviewed 11 studies including 181 745 patients. They concluded that the overall prevalence of radiocontrast media HSRs in the Asia Pacific regions seems to be similar to those on other continents.^17^

### Limitations

Some limitations need to be acknowledged: (1) In Phase-IV-Analysis, 7200, 92 821, and 5584 patients for sex, race, and region/country, respectively, had to be excluded upfront from FAS because of missing key data (Figure 1). In GPV-Analysis, out of >333 million administrations, only 78.72 million and 118.56 million were eligible for the sex and region/country evaluation, respectively. Race could not be analysed at all as race is not captured by GPV and was not available in market research. (2) Serious cases reported in the Phase-IV-Analysis of the 4 observational studies are necessarily also included in the GPV database. However, these were just 905 cases in 17 841 cases (Table 5). (3) In observational studies and even more in pharmacovigilance databases, underreporting cannot be ruled out.^24^ (4) This study did not analyse if iopromide was used as a substitute LOCM in order to minimize recurrent HSR, a topic currently in scientific discussion.^37^^,^^38^

## Conclusion

Women showed a slightly higher risk for HSRs to iopromide than men. This is considered an LOCM class effect. Evidence of an impact of race was not found. HSR reporting varied slightly by region/country.

## Data Availability

The datasets generated or analysed during the study are available from the corresponding author on reasonable request.
